# Sources, perceived usefulness and understanding of information disseminated to families who entered home quarantine during the H1N1 pandemic in Victoria, Australia: a cross-sectional study

**DOI:** 10.1186/1471-2334-11-2

**Published:** 2011-01-04

**Authors:** Anne M Kavanagh, Rebecca J Bentley, Kate E Mason, Jodie McVernon, Sylvia Petrony, James Fielding, Anthony D LaMontagne, David M Studdert

**Affiliations:** 1 Centre for Women's Health, Gender and Society, Melbourne School of Population Health, The University of Melbourne, Melbourne, Victoria, Australia; 2 Vaccine & Immunisation Research Group, Murdoch Children's Research Institute and Melbourne School of Population Health, The University of Melbourne, Melbourne, Victoria, Australia; 3 Victorian Infectious Diseases Reference Laboratory, Melbourne, Victoria, Australia; 4 National Centre for Epidemiology and Population Health, The Australian National University, Canberra, Australian Capital Territory, Australia; 5 Victorian Government Department of Health, Melbourne, Victoria, Australia; 6 McCaughey Centre, Melbourne School of Population Health, The University of Melbourne, Melbourne, Victoria, Australia; 7 Centre for Health Policy, Programs and Economics, Melbourne School of Population Health, The University of Melbourne, Melbourne, Victoria, Australia

## Abstract

**Background:**

Voluntary home quarantine of cases and close contacts was the main non-pharmaceutical intervention used to limit transmission of pandemic (H1N1) 2009 influenza (pH1N1) in the initial response to the outbreak of the disease in Australia. The effectiveness of voluntary quarantine logically depends on affected families having a clear understanding of what they are being asked to do. Information may come from many sources, including the media, health officials, family and friends, schools, and health professionals. We report the extent to which families who entered home quarantine received and used information on what they were supposed to do. Specifically, we outline their sources of information; the perceived usefulness of each source; and associations between understanding of recommendations and compliance.

**Methods:**

Cross-sectional survey administered via the internet and computer assisted telephone interview to families whose school children were recommended to go into home quarantine because they were diagnosed with H1N1 or were a close contact of a case. The sample included 314 of 1157 potentially eligible households (27% response rate) from 33 schools in metropolitan Melbourne. Adjusting for clustering within schools, we describe self-reported 'understanding of what they were meant to do during the quarantine period'; source of information (e.g. health department) and usefulness of information. Using logistic regression we examine whether compliance with quarantine recommendations was associated with understanding and the type of information source used.

**Results:**

Ninety per cent understood what they were meant to do during the quarantine period with levels of understanding higher in households with cases (98%, 95% CI 93%-99% vs 88%, 95% CI 84%-91%, P = 0.006). Over 87% of parents received information about quarantine from the school, 63% from the health department and 44% from the media. 53% of households were fully compliant and there was increased compliance in households that reported that they understood what they were meant to do (Odds Ratio 2.27, 95% CI 1.35-3.80).

**Conclusions:**

It is critical that public health officials work closely with other government departments and media to provide clear, consistent and simple information about what to do during quarantine as high levels of understanding will maximise compliance in the quarantined population.

## Background

In the absence of an effective vaccine, social distancing is a key strategy for preventing the spread of emerging, potentially serious, infectious respiratory diseases [1]. Voluntary home quarantine of cases and close contacts was the main non-pharmaceutical intervention used to limit transmission of pandemic (H1N1) 2009 influenza (pH1N1) in the initial response to the outbreak of the disease in Australia. The Australian Government's management plan for pandemic influenza recommended school and classroom closures to reduce the early spread of the virus [2]. School closures and home quarantine became a key strategy during the 'contain phase' of the outbreak (22 May - 2 June 2009) [3], particularly in Victoria, because the majority of Australia's HIN1 cases occurred among school-aged children in that state [4-6].

The effectiveness of voluntary quarantine logically depends on affected families having a clear understanding of what they are being asked to do. Typically, however, the conditions are not conducive to conveying clear messages. As outbreaks unfold quickly, information tends to come from many sources, including the media, health officials, family and friends, schools, employers and health professionals. In previous epidemics, the accuracy, clarity, and usefulness of this information have been shown to vary greatly [7]. Two Australian studies of quarantine compliance included a study of Western Australian school children [8] and a national study that reported intention to comply among unaffected individuals [9]; neither of these studies reported on understanding of quarantine recommendations or information sources used. In fact we could not identify any published studies that have reported the sources of information, understanding of recommendations and compliance.

We conducted a cross-sectional survey of Victorian households with children who were placed in voluntary home quarantine during the contain phase of the pH1N1 outbreak. The survey probed participants' understanding of the quarantine recommendations, the information sources used to gain this understanding, and the perceived usefulness of those sources. We also analysed whether these factors were associated with levels of compliance among families. Our goal was to inform the design and implementation of communication strategies around quarantine in future pandemics.

## Methods

### Study Environment

The first Australian case of pH1N1 was identified on 8 May 2009 [10]. Two weeks later, Victoria's first case was identified, a nine year-old boy who had recently returned from the United States [4]. In the 12-day contain phase that followed, cases and their immediate family members and close contacts were asked to go into home quarantine. Quarantined persons were expected to have no contact with non-household members and were treated with Oseltamivir for ten days. Cases were asked to stay in quarantine for seven days after the onset of symptoms. Contacts—defined as individuals who spent more than four hours in the same room as the confirmed case, or were within one metre of the confirmed case for more than 15 minutes—were asked to stay in home quarantine for seven days from last date of exposure to the case (Department of Health Victoria quarantine guidelines, 4 June 2009).

The trigger for closure of mainstream schools was two or more confirmed cases in separate classes. However only cases and fellow students who met the definition of contacts were placed in home quarantine; other students in closed schools were merely asked to limit their outside activities (Department of Health Victoria quarantine guidelines, 4 June 2009). The policy at special developmental schools (SDS) differed from mainstream schools: a confirmed case triggered home quarantine for the entire student body.

### Sample

**Figure 1:**
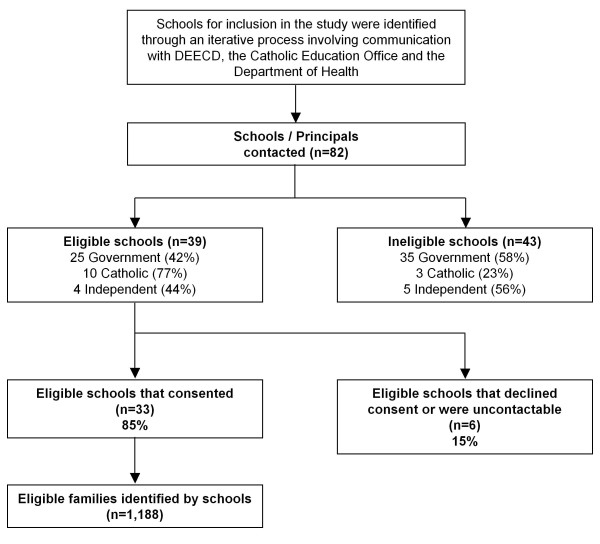
**Recruitment of parents whose school children were recommended to go into home quarantine (May 22nd until June 2nd, 2009)**.

We identified affected households through schools. During the outbreak, the Victorian Departments of Education and Early Child Development (DEECD) and Health and the Catholic Education Office were actively involved in visiting schools, identifying cases and determining the need for quarantine. Each of these agencies held separate but incomplete information on closure and quarantine activities in schools. After pooling this information, we approached Principals at 82 schools that were known or suspected to have implemented closures and asked children to enter quarantine (Figure 1). For Catholic Schools, the DEECD information was reconciled with the information held by the Catholic Education Office before schools were approached. As a consequence of this a smaller proportion of Catholic schools were considered ineligible after schools were contacted directly (23% of Catholic Schools and 58% of government schools). We posed two eligibility questions to the Principals, namely, did the school have (1) classes closed during the contain phase of the outbreak? and (2) children who were asked to go into home quarantine?

Three Principals did not respond to our approaches, three declined to participate, and 39 schools did not meet the eligibility criteria (i.e. the Principals answered "no" to one or both of the eligibility questions). Of the rest, 33 Princpals agreed to facilitate the conduct of the survey resulting in an eligible school participation rate of 85%.

We worked with staff of these 33 schools to identify 1,188 families who experienced quarantine. School staff used a mix of information to identify these families, including enrolment records, class lists and documentation of which classes and students had been asked to enter quarantine. Our research team guided the school staff through the process of assembling and reviewing this information, but we did not have contact with data identifying students or families, either at this stage or during subsequent administration of the survey.

The study was approved by the ethics committee at the University of Melbourne (0932293) and the DEECD and the Catholic Education Office granted us permission to approach schools to conduct the survey.

### Survey Questionnaire

The questionnaire had several modules. One module gathered demographic details about the family, including household composition, education, employment, housing and income. Another module elicited information on whether each member of the household: was a contact or case (defined as having a pH1N1 diagnosis confirmed by a laboratory or medical practitioner); received Oseltamivir for treatment or prophylaxis; and complied with quarantine.

Another module, the focus of this paper, asked participants whether they understood what their family was being asked to do during the quarantine period, where they obtained information on what to do, and how useful various sources of such information proved to be. Specifically, participants were asked to rate on a five-point scale ranging from strongly agree to strongly disagree the extent to which they agreed or disagreed with the statement *"At the time of the quarantine measures I understood what my family was being asked to do"*. Participants were also asked where they obtained "*information about what you were supposed to do in quarantine" *with the following response options: health department (which might refer to state or federal government); school; general practitioner (GP)/other health care provider; family/friends; media (newspaper/tv) and other. Multiple responses were possible. Finally, participants were asked to rate the usefulness of each information source.

For analytical purposes, we collapsed the gradations of understanding into a binary variable (strongly agree/agree vs. neither agree nor disagree/disagree/strongly disagree).

We defined a household as compliant with quarantine recommendations if they met all of the following criteria: (1) All quarantined members of the household stayed at home for most of each day; (2) No quarantined household members visited public places with lots of other people (excluding visits to health practitioners); (3) No adults from other households visited the home for ≥15 minutes; (4) Quarantined children did not mix with children from another household for ≥15 minutes; and (5) Any childcare was only provided by adults living in the household.

### Survey Administration

The survey was administered during November and December 2009. School staff mailed letters to the parents in eligible families inviting them to participate. The letter presented two options: an internet address at which parents could complete the questionnaire online and a telephone number to ring to complete it via a Computer Assisted Telephone Interview (CATI). The survey was offered in English only. The letter also included a unique 8-digit identification number which enabled access to the website and CATI. This number allowed us to identify the school(s) and home class(es) of each family's child(ren), but revealed no other identifying information.

School staff mailed two reminder letters. To boost response rates and recognise the effort involved for participating families and schools we contributed $AU20 to the school for the purchase of educational resources for each completed questionnaire and all families received a movie voucher valued at AU$10.30 with the second reminder letter.

Eight letters were returned-to-sender and 23 parents responded indicating that they did not have a school-aged child who had been placed in home quarantine. This left an in-scope sample of 1,157. We received 314 responses yielding a household participation rate of 27%. Missing data on key questions related to the information sources reduced our analysable sample for this study to 297 families.

### Analysis

All analyses were conducted in Stata 11.0 (STATA Corp, College Station, TX). We calculated proportions for each of the variables of interest (household understanding of quarantine requirements, and use and perceived usefulness of information sources) and stratified these proportions by whether the households had a case or contacts only. To account for within-school clustering, we used logistic regression (using Stata's cluster command) and post-estimation commands to generate proportions, 95% confidence intervals and p-values.

We also used logistic regression, again adjusting for within-school clustering, to examine whether compliance with quarantine recommendations was associated with understanding of quarantine recommendations and the type of information source used. The types of information were grouped into official sources (health department and schools) and unofficial sources (media, family and friends and health care providers). We postulated that these relationships may be confounded by two variables—whether a household had a case or contacts only, and level of parental education—and so included these as covariates. However, because adjustment for these variables did not change the size and significance of the coefficients of interest, we report unadjusted estimates.

## Results

### Sample characteristics

Seventeen per cent of participants reported having had a confirmed case of pH1N1 in their household (Table 1). Seventy-six per cent of the quarantined children attended government schools, 15% attended Catholic schools and 9% attended Independent schools. Forty-one per cent of the children were in primary school, 35% were in secondary school and 24% were in Special Development Schools.

**Table 1:** Demographic characteristics of sample (n = 297)

	n	%
**Sex of respondent**		
Female	254	85.5
**Age of oldest child**		
Under 12	145	49.0
**Number of children in home quarantine**		
Two or more	46	15.5
**Households with a case**		
Case in household	51	17.2
School sector*		
Government	226	76.1
Catholic	45	15.2
Independent	26	8.8
School level*		
Primary	123	41.4
Primary/Secondary	1	0.3
Secondary	103	34.7
Special Development	70	23.6
**Household composition**		
Single parent, one child	12	4.0
Single parent, 2+ children	24	8.1
Couple, one child	40	13.5
Couple, 2+ children	221	74.4
**Highest level of parental education**		
Bachelor degree or higher	155	52.5

* refers to the school through which the household was contacted

### Understanding of quarantine recommendations

Ninety per cent (266/297) of participants understood what they were meant to do during the quarantine period. This proportion was significantly higher in households with cases than in households with contacts only (98%, 95% CI 93%-99% vs 88%, 95% CI 84%-91%, P < 0.001).

### Information sources

Nearly 90% of parents received information about quarantine from the school and 63% obtained information from the health department (Table 2). The next most common information source was the media (44%). Overall, most families used multiple sources of information; only one household reported that they did not use any sources. 24% used only one source, 32% used two, and 44% used three or more.

**Table 2:** Information sources used by parents whose children were placed in home quarantine

	% who obtained information from source					
	
	Total		Case in household		No case in household	
Information Source	n	%	%	95% CI	%	95% CI
School	257	86.5	51.0	(38.1, 63.7)	93.9	(89.8, 96.4)
Health Department	187	63.0	80.4	(64.2, 90.4)	59.3	(49.1, 68.8)
Media (newspaper/TV)	132	44.4	54.9	(42.4, 66.8)	42.3	(38.3, 46.4)
GP/other healthcare provider	84	28.3	58.8	(46.1, 70.5)	22.0	(15.6, 30.0)
Family/friends	51	17.2	13.7	(7.7, 23.1)	17.9	(14.4, 22.0)
Other	23	7.7	6.0	(2.4, 14.0)	8.1	(4.8, 13.3)

A minority of participants reported using official sources only (n = 120, 40%). The majority (n = 172, 58%) used both official and unofficial sources of information. Only five households did not use any official sources.

There was some evidence that case households and contact-only households received their information from different sources. Case households were more likely to receive information from the health department (80%, 95 CI 64%-90% vs 59%, 95% CI 49%-69%, P = 0.015) and were less likely to receive their information through schools (51%, 95% CI 38%-64% vs 94% 95% CI 90%-96%, P < 0.001).

### Perceptions of usefulness of information

Approximately two-thirds of participants reported that they found the information from the health department, schools and health service providers useful or extremely useful, whereas only 38% gave media sources this rating (Table 3). There were no significant differences in usefulness ratings between case households and contact-only households.

**Table 3:** Usefulness of information sources in H1N1 pandemic in Victoria, Australia

	% useful or extremely useful					
	**Total**		**Case in household**		**No case in household**	
**Information Source**	**n**	**%**	**%**	**95% CI**	**%**	**95% CI**

Health Department	127	68.3	60.0	(46.1, 72.4)	70.3	(64.7, 75.5)
School	168	65.9	68.0	(49.5, 82.2)	65.9	(57.0, 73.9)
GP/other healthcare provider	51	63.0	71.4	(55.4, 83.4)	57.7	(43.9, 70.3)
Media (newspaper/TV)	51	38.6	48.1	(33.7, 62.7)	36.5	(27.8, 46.2)
Family/friends	16	32.0	42.9	(23.1, 65.2)	30.2	(18.5, 45.3)
Other	17	73.9	100.0	(29.2, 100.0)*	70.0	(50.5, 89.5)

*one-sided confidence interval

### Relationship between understanding, information and compliance

Fifty-three per cent of participants reported full compliance with quarantine recommendations within their household. Of the 90% of respondents who reported understanding what they were meant to do during quarantine, 55% (n = 147) reported full compliance. In contrast, full compliance was only reported by 35% (n = 11) of the minority who did not report that they understood what they were meant to do. Compliance was higher in the households that reported understanding what they were meant to do during the quarantine period (Odds Ratio 2.27, 95% CI 1.35-3.80). There were no differences in the odds of compliance between households that used official sources of information only compared to those that used both official and unofficial sources (Odds Ratio 1.00, 95% CI 0.69-1.44). (The five households that did not use any official sources were excluded from this analysis.)

## Discussion

Families with school-children who entered quarantine during Victoria's pH1N1 relied heavily on official sources of information, particularly schools and the health department. Troublingly, one third of families who used these sources did not find them useful in gaining an understanding of what they were supposed to do during quarantine. The media was the next most relied upon source although nearly 60% of families did not find this source illuminating. Our findings also suggest that the stakes associated with lack of comprehension are high, as the odds of compliance were more than twice as high among families who understood the home quarantine recommendations.

Liaising closely with a range of different media (such as print, television and internet) is critical, however media interests may not be congruent with optimal public health policy [7]. The fact that 44% of families in our study turned to the media as a source of information during the contain phase of the pandemic, but a minority found media information useful, indicates that there is much room for improvement in coordinating the messages coming from official and non-official sources. In future pandemics, which may be more severe and of longer duration than pH1N1, Australian government officials will need to work more closely with the media to provide accurate, easy-to-understand information on social distancing measures and other preventative strategies.

As most Australian cases occurred in Victorian schoolchildren, who became the chief target of preventive measures to reduce spread of pH1N1, our study provides valuable insights into information sources, understanding and compliance among families most affected by an emerging pandemic. However, the study has some limitations. Due to ethical and privacy issues the survey was not conducted until November and December 2009 (six months after the home quarantine measures had been implemented), introducing the potential for recall bias. Another potential problem relates to the way in which the question about information sources was asked, whereby we do not know how respondents who used the media to obtain information from health department would have answered. It is possible that they ticked health department, media or both. A European study found that national and international public health authorities were by far the leading source of information in articles in the media on H1N1 influenza in the early stage of the pandemic [11]. If the same pattern was observed in Australia then it is likely that families accessed information from the health department through the media.

We had a relatively low response rate, although it is close to those achieved in similar studies that had much smaller population samples [12-14]. We had to administer the survey through schools due to privacy concerns and this is likely to have contributed to our low response rate. It also likely that response rates were low because most of the schools were located in the Northern Metropolitan region of Melbourne, an area that has higher levels of disadvantage and a greater proportion of households that speak a language other than English at home (http://www.abs.gov.au/websitedbs/D3310114.nsf/home/Census+data; accessed August 10/2010). In addition, the internet was the main mode of survey administration which may have reduced access for disadvantaged groups. To the extent that this type of response bias occurred, it is likely to make our estimates of the understanding and perceived usefulness of quarantine information among affected families higher than might be the case in all families affected by quarantine. It is possible that non-responders were less interested in H1N1 or health issues in general and that their understanding of information and the sources of information used may differ from responders. Without more information it is not possible to know how non-response bias might have affected our estimates of understanding of quarantine recommendations or the source of information.

## Conclusions

Our findings reinforce the importance of providing clear messages about home quarantine and suggest that success in this area is likely to have a substantial impact on compliance. Closer attention to how government recommendations about quarantine are presented is needed, as one third of the sample reported that information obtained from these sources was not useful. Qualitative interviews with affected households might provide further insights into how the provision of this information could be improved. The quality and clarity of information from unofficial sources, particularly the media, is also important, recognising that nearly half the households in our study used media sources but two-thirds of them did not find this information useful. Coordination between the major information sources is also essential: government should work closely with the media to facilitate consistent messages, including responsible and accurate reporting of quarantine recommendations and other social distancing measures. Finally, future pandemic management may benefit from the implementation of a process to monitor in real time how communication messages are being received, thereby allowing timely analyses and amendments rather than relying on collecting information many months after the event.

The relatively benign nature of the recent pH1N1 in Australia probably prevented shortcomings in communication and outreach activities from causing serious harm. However, the next pandemic may be crueler: it may cause more serious morbidity and mortality, last longer, and necessitate the issuing of a range of recommendations over time to guide public action. Under those conditions, weaknesses in communication strategies will be exposed and may cost lives.

## Competing interests

The authors declare that they have no competing interests.

## Authors' contributions

AK conceived of the study and drafted the manuscript; RB contributed to the conception of the study and design and advised on analysis; KM developed the analytic plan and conducted the analyses; JM, JF, AL and DS contributed to the conception of the study and design; and SP contributed to the design of the survey and was responsible for its implementation. All authors contributed to the drafting of the manuscript and have read and approved the final manuscript.

## Pre-publication history

The pre-publication history for this paper can be accessed here:

http://www.biomedcentral.com/1471-2334/11/2/prepub
